# Efficacy and safety of low-molecular-weight heparin after knee arthroscopy: A meta-analysis

**DOI:** 10.1371/journal.pone.0197868

**Published:** 2018-06-21

**Authors:** Hai-Feng Huang, Jia-Liang Tian, Xian-Teng Yang, Li Sun, Ru-Yin Hu, Zhi-Hui Yan, Shan-Shan Li, Quan Xie, Xiao-Bin Tian

**Affiliations:** 1 Medical College, Guizhou University, Guiyang, Guizhou, China; 2 Department of Orthopaedics, Guizhou Provincial People’s Hospital, Guiyang, Guizhou, China; 3 Department of Anesthesiology, Guizhou Provincial People’s Hospital, Guiyang, Guizhou, China; 4 College of Big Data and Information Engineering, Guizhou University, Guiyang, Guizhou, China; Universite de Bretagne Occidentale, FRANCE

## Abstract

**Background:**

Venous thromboembolism (VTE) is considered a potentially serious complication of knee arthroscopy and leads to conditions such as deep venous thrombosis (DVT) and pulmonary embolism (PE). Low-molecular-weight heparin (LMWH) is widely employed in knee arthroscopy to reduce perioperative thromboembolic complications. However, the efficacy and safety of LMWH in knee arthroscopy remains unclear.

**Methods:**

Seven randomized controlled clinical trials on LMWH in knee arthroscopy were identified and included in this meta-analysis. The main outcomes of the effectiveness (prevention of DVT and PE) and complications (death, major bleeding, and minor bleeding) of LMWH in knee arthroscopic surgery were assessed using Review Manager 5.3 software.

**Results:**

The meta-analysis indicated that LMWH prophylaxis comprised 79% of asymptomatic DVT. No association was found in symptomatic VTE (RR: 0.90; 95% confidence interval [CI]: 0.39–2.08; P = 0.80), symptomatic DVT (RR: 0.79; 95% CI: 0.28–2.23; P = 0.66), symptomatic PE (RR: 1.36; 95% CI: 0.37–4.97; P = 0.64) and major bleeding (RR: 0.70; 95% CI: 0.12–3.95; P = 0.68) risk during LMWH prophylaxis were identified. Death was not reported in these studies. Moreover, there was a lower incidence of minor bleeding (RR: 0.64; 95% CI: 0.49 to 0.83; P = 0.001) in the control group than in the LMWH group.

**Conclusion:**

Compared with the control group, the group treated with LMWH after knee arthroscopy was no association in reducing the symptomatic VTE rate, symptomatic DVT rate or symptomatic PE rate. The symptomatic VTE rate was 0.5% (11/2,166) in the LMWH group versus 0.6% (10/1,713) in the control group. Although the limitations of this meta-analysis cannot be ignored, the results of our study show that LMWH after knee arthroscopy is ineffective. We recommend that LMWH should not be routinely provided for knee arthroscopy.

**Trial registration:**

ClinicalTrials.gov NCT03164746

## Introduction

Knee arthroscopy has become one of the most common surgical methods for the knee. Many patients suffering from knee diseases experience satisfactory curative effects using this method. However, severe clinical symptoms, including deep venous thrombosis (DVT) and pulmonary embolism (PE), have occurred during the postoperative period. Venous thromboembolism (VTE) after knee arthroscopy is not an uncommon complication and is an important health problem. Following arthroscopic knee surgery, patients are at an increased risk of complications associated with VTE (i.e., DVT or PE). [1] Complications of VTE include PE, which may be fatal. VTE is a disease that causes a major health burden in different countries and regions. [2] Therefore, effective postoperative prevention of VTE appears to be critical to operative success. Anticoagulants are the main prophylaxis for VTE. Studies have indicated that patients undergoing knee arthroscopy receive effective prophylaxis of VTE using Low-molecular-weight heparin (LMWH). [3–7] Furthermore, a recent meta-analysis indicated that LMWH has the potential to prevent thromboembolic events in non-major orthopaedic settings compared with no treatment. [8] However, a recent randomized controlled trial (RCT) showed that the use of LMWH after knee arthroscopic surgery is not effective for preventing symptomatic VTE. [9] Thus, the efficacy of LMWH in knee arthroscopy remains controversial.

LMWHs, such as tinzaparin, dalteparin and enoxaparin, have high potentials for patient administration and bioavailability. [10–11] VTE prophylaxis with LMWH is indicated for patients after arthroscopic surgery, and some studies indicate that patients received beneficial effects; however, disadvantages also exist with LMWH. The potential risk of bleeding following the use of LMWH is unavoidable. Moreover, no meta-analysis has investigated the safety of LMWH in patients after knee arthroscopy.The debate regarding whether the use of LMWH has a preventive effect after knee arthroscopy is ongoing. The purpose of this meta-analysis is to assess the efficacy and safety of LMWH in arthroscopic surgery of the knee.

## Methods

### Search strategy

All eligible studies were obtained from PubMed, EMBASE and the Cochrane Library. The following search terms, including keyword or entry terms, were used without limitations through September 2017: ‘arthroscopy OR arthroscopic OR arthroscopically’ AND ‘Heparin, Low-Molecular-Weight OR Heparin, Low Molecular Weight OR LMWH OR Low Molecular Weight Heparin OR Low-Molecular-Weight Heparin’. We manually screened the relevant review articles in the reference lists.

### Selection criteria

The following trials were identified and included in this study: (1) patients treated with knee arthroscopy, (2) patients who received LMWH after knee arthroscopy, (3) outcomes included efficacy (DVT, PE) and safety (major bleeding, minor bleeding), and (4) studies were randomized controlled clinical trials.

### Data extraction

For each study, two independent authors, using a standardized data extraction form, extracted the following data: authors, year of publication, study quality, intervention, population, patient characteristics, and outcomes. We accepted the authors’ definitions of the results and did not reclassify the events. The efficacy endpoint was VTE (symptomatic VTE, asymptomatic DVT, symptomatic DVT and symptomatic PE) in the studies. DVT confirmation was required by venography or compression ultrasound, including asymptomatic and symptomatic DVT. Symptomatic PE confirmation was required by pulmonary angiography, ventilation-perfusion lung scanning or helical computed tomography. The primary safety endpoint was defined as major bleeding or all-cause death. The secondary safety endpoint was defined as a minor bleeding event.

### Quality assessments

To evaluate the risk of bias of the selected studies, the Cochrane Collaboration tool was used. For each study, individual team members judged the risk of bias in a given study and determined it to be “low”, “high”, or “unclear”. Each disagreement was referred to a third team member.

### Statistical analysis

All statistical data were entered into Review Manager version 5.3 software (Copenhagen: The Nordic Cochrane Centre, The Cochrane Collaboration). Random- or fixed-effects models were used to calculate the summary risk based on the heterogeneity levels. Heterogeneity between studies was estimated using the *I*^2^ statistic. In the heterogeneity levels across studies, *I*^2^ measured the quantitative inconsistency. *I*^2^ values from 25% to 50% showed low heterogeneity, values from 50% to 75% exhibited moderate heterogeneity, and *I*^2^ values >75% showed high heterogeneity. An *I*^2^ value >0.50 and a P-value for heterogeneity <0.10 indicated significant heterogeneity.

## Results

### Literature search and study characteristics

**Fig 1:**
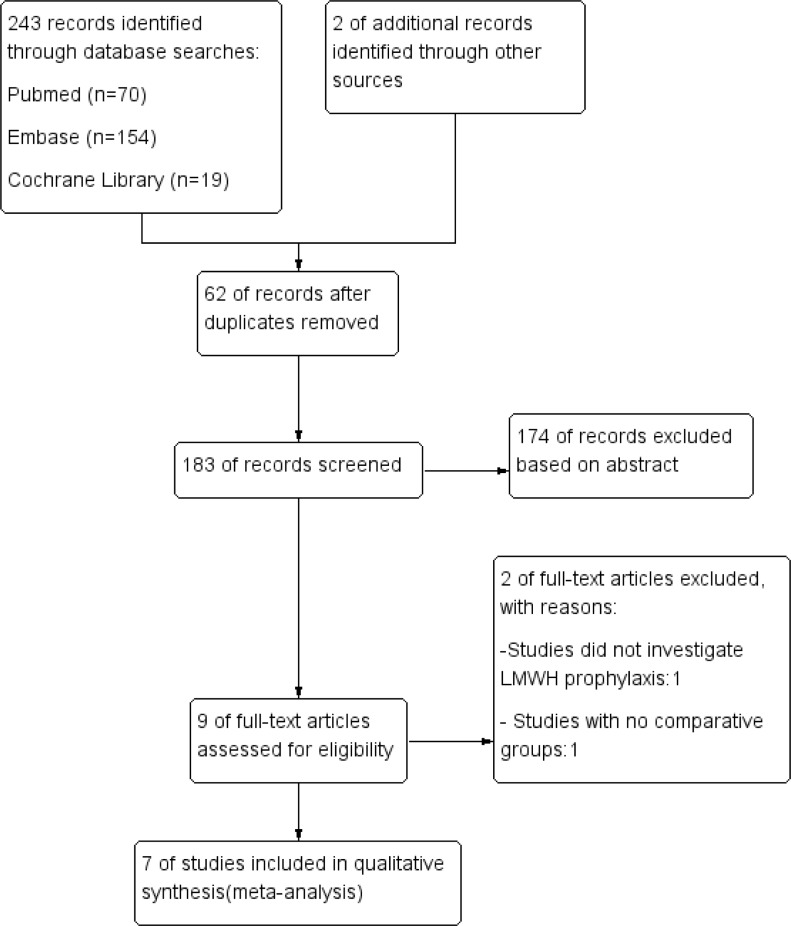
Flowchart of the screened, excluded, and analysed publications.

**Fig 2:**
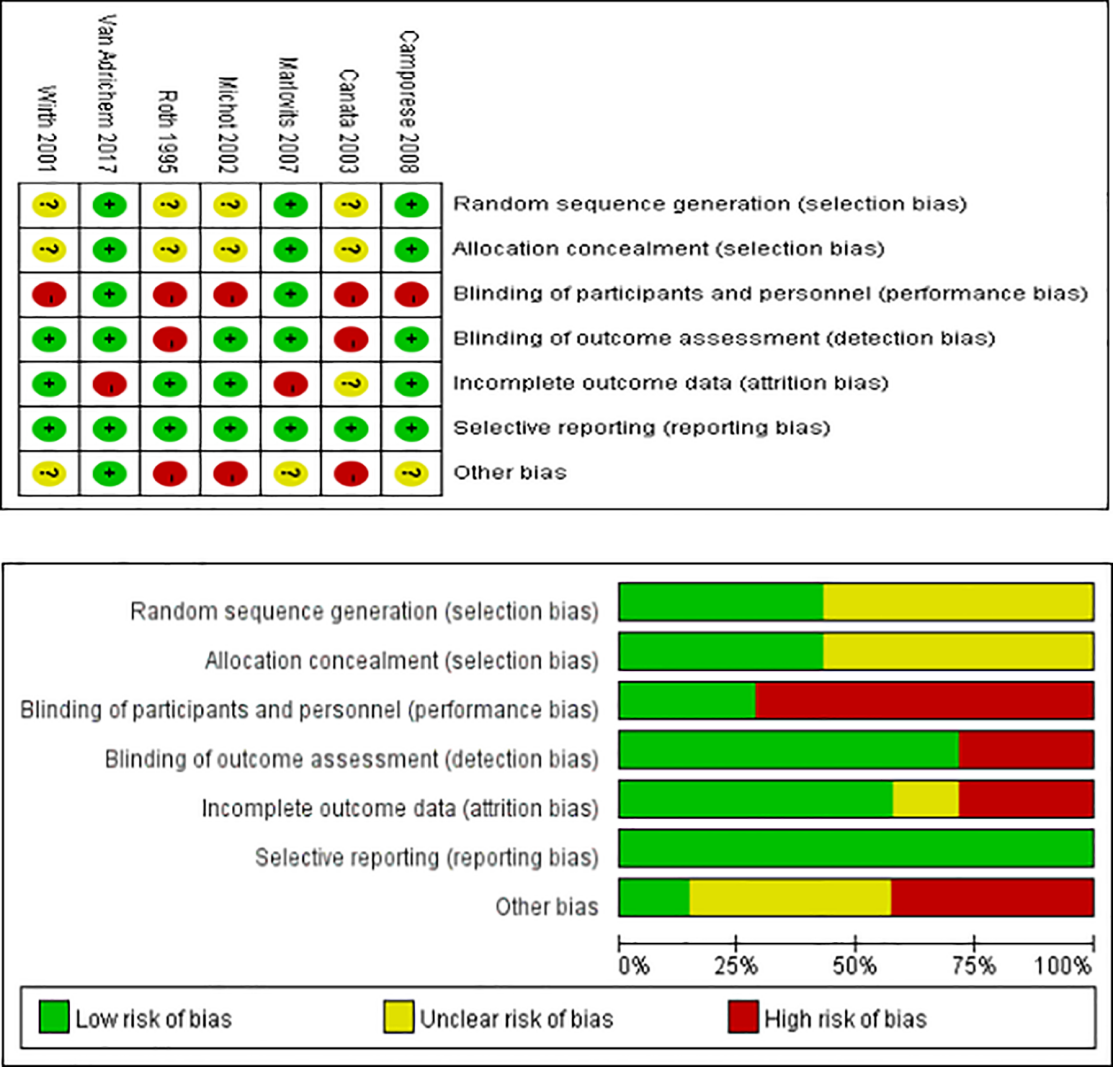
Risk of bias of the selected studies according to the Cochrane Collaboration tool. Panel A: Risk of bias graph: judgement regarding each risk of bias item presented as percentages across all studies. Panel B: Risk of bias summary: judgement regarding each risk of bias item for each study. A (+), indicates a low risk of bias; a (-), indicates a high risk of bias; and a (?), indicates an unclear risk of bias. All assessments were determined by consensus of the two independent authors.

The details of the search strategy are shown in Fig 1. According to the search strategy and inclusion criteria, 245 articles were identified through the initial search, of which 7 randomized controlled trials [3–7,9,12] comprising 3,879 patients (2,166 individuals in the LMWH group and 1,713 individuals in the control group) were included in our meta-analysis. The Cochrane Collaboration tool was used to evaluate the quality of the eligible studies (Fig 2). Table 1 shows the main characteristics of the 7 studies.

**Table 1:** Characteristics of the 7 selected clinical studies.

Study	Study style	Medicine group	LMWH type, dose and average use time	No. of patients	Sex M/F	Age, year	BMI, kg/m^2^	Average operation time (min)	Tourniquet used (patients)	Average tourniquet inflation time (min)	Primary outcome event and classification (asymptomatic DVT; diagnosed by compression ultrasound or venography)	Secondary outcome event and classification (symptomatic VTE excluding distal VTE; diagnosed by clinical assessment)
**Roth** **1995[3]**	**RCT**	**LMWH**	**0.3 ml Fraxiparine; 4 days after surgery**	**61**	**NA**	**NA**	**NA**	**NA**	**NA**	**NA**	**0**	**1**
		**None**		**61**	**NA**	**NA**	**NA**	**NA**	**NA**	**NA**	**4**	**1**
**Wirth** **2001[4]**	**RCT**	**LMWH**	**Reviparin; 1,750 IU; 7 to 10 days**	**117**	**81/36**	**37.6**	**The average height was 176 cm, and the body weight was 80.7 kg.**	**39**	**117**	**42**	**1**	**0**
		**None**		**122**	**98/24**	**38.5**	**The average height was 177 cm, and the body weight was 81.1 kg**	**29**	**122**	**38**	**2**	**0**
**Michot** **2002[5]**	**RCT**	**LMWH**	**Dalteparin; 2,500 IU if weight <70 kg, 5,000 IU if >70 kg; 30 days**	**66**	**40/26**	**42.0**	**26.2**	**42.3**	**44**	**NA**	**1**	**0**
		**None**		**64**	**46/18**	**46.5**	**27.8**	**38.2**	**40**	**NA**	**10**	**0**
**Canata** **2003[12]**	**RCT**	**LMWH**	**Enoxaparin; no dose specified; 6 days**	**18**	**12/6**	**29.6**	**NA**	**NA**	**NA**	**NA**	**NA**	**0**
		**None**		**18**	**13/5**	**32.5**	**NA**	**NA**	**NA**	**NA**	**NA**	**0**
**Marlovits 2007[6]**	**RCT**	**LMWH**	**Enoxaparin; 40 mg; 20 days**	**72**	**45/27**	**41.7% of patients aged >30 years**	**NA**	**50% patients (length of operation >2 hours)**	**NA**	**NA**	**2**	**0**
		**Placebo**		**68**	**37/31**	**41.2% of patients aged >30 years**	**NA**	**50% patients (length of operation >2 hours)**	**NA**	**NA**	**25**	**3**
**Camporese 2008[7]**	**RCT**	**LMWH**	**Nadroparin; 3800 IU; 7 days or 14 days**	**1101**	**679/422**	**42.1**	**25.4**	**NA**	**1101**	**38**	**15**	**5**
		**Graduated compression stockings**		**660**	**412/248**	**42.3**	**25.5**	**NA**	**660**	**36**	**17**	**3**
**Van Adrichem 2017[9]**	**RCT**	**LMWH**	**Nadroparin or dalteparin; 2500 IU of dalteparin or 2850 IU of nadroparin were used for patients who weighed** **100 kg or less, and a double dose was used for patients who weighed more** **than 100 kg; 8 days**	**731**	**414/317**	**48.1**	**27.1**	**26**	**688**	**NA**	**NA**	**5**
		**None**		**720**	**396/324**	**49.1**	**26.8**	**26**	**673**	**NA**	**NA**	**3**

BMI = body mass index; NA = not available

### Efficacy of LMWH

**Fig 3:**
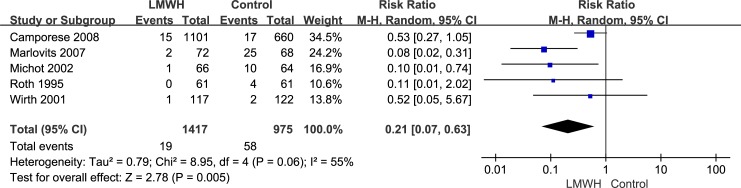
LMWH versus control. Asymptomatic deep vein thrombosis during follow-up.

**Fig 4:**
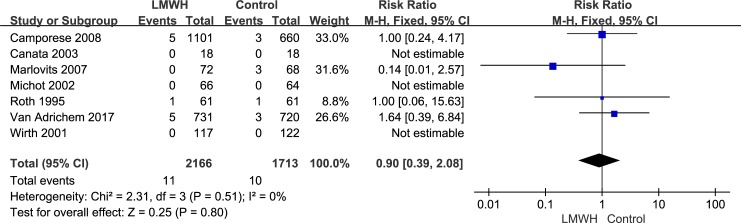
LMWH versus control. Symptomatic venous thromboembolism during follow-up.

**Fig 5:**
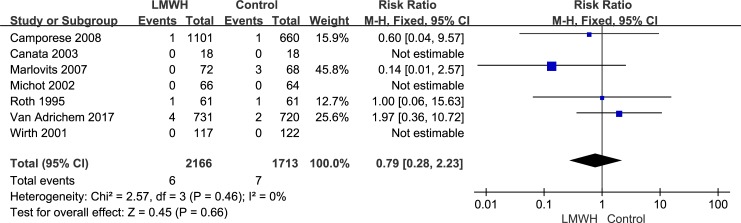
LMWH versus control. Symptomatic deep vein thrombosis during follow-up.

**Fig 6:**
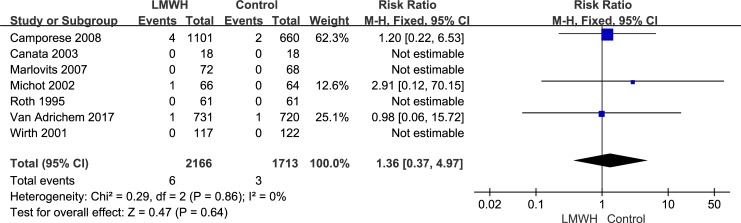
LMWH versus control. Symptomatic pulmonary embolism during follow-up.

All seven randomized controlled trials, which included 3,879 patients, reported the efficacy (symptomatic VTE, symptomatic DVT and symptomatic PE) of LMWH use after knee arthroscopic surgery. However, only five randomized controlled trials, which included 2,392 patients, reported the efficacy of asymptomatic DVT. We used the random-effects model to combine asymptomatic DVT data according to notable heterogeneity (*I*^2^ value = 55%). Compared with the control group, after arthroscopic knee surgery, the incidence of asymptomatic DVT in the LMWH group was lower (RR: 0.21; 95% CI: 0.07–0.63; P = 0.005, Fig 3). We used the fixed-effects model to combine symptomatic VTE data according to lower significant heterogeneity (*I*^2^ value = 0%). Compared with the control group, after arthroscopic knee surgery, the incidence of symptomatic VTE in the LMWH group was not significantly different (RR: 0.90; 95% confidence interval [CI]: 0.39–2.08; P = 0.80, Fig 4). The fixed-effects model was used to combine symptomatic DVT data because heterogeneity was not evident (*I*^2^ value = 0%). The symptomatic DVT rate was 0.3% (6/2,166) in the LMWH group versus 0.4% (7/1,713) in the control group. In the overall data, the symptomatic DVT rate in the control group was not significantly different from that in the LMWH group (RR: 0.79; 95% CI: 0.28–2.23; P = 0.66, Fig 5). As a result of the lower heterogeneity of the data (*I*^2^ value = 0%), we used the fixed-effects model to combine the symptomatic PE data. The meta-analysis demonstrated that the symptomatic PE rate was not significantly affected by LMWH treatment after knee arthroscopy (RR: 1.36; 95% CI: 0.37–4.97; P = 0.64, Fig 6).

### Safety of LMWH

**Fig 7:**
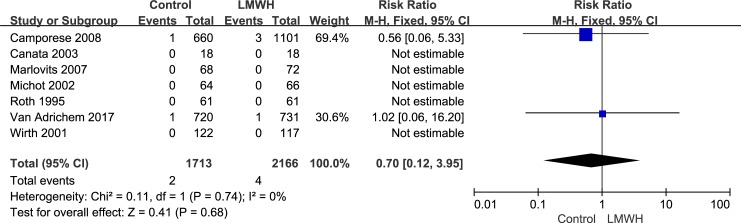
LMWH versus control. Major bleeding during follow-up.

**Fig 8:**
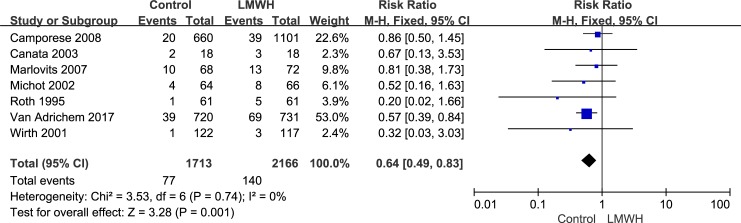
LMWH versus control. Minor bleeding during follow-up.

The studies included 3,879 patients and reported the safety of LMWH, including the side effects of major bleeding and minor bleeding after knee arthroscopy, in the two groups. The fixed-effects model was used to combine the major bleeding data because of its low heterogeneity (*I*^2^ value = 0%). In the combined data, this model showed no significant difference between the LMWH and control groups with respect to major bleeding during the postoperative period (RR: 0.70; 95% CI: 0.12–3.95; P = 0.68, Fig 7). The major bleeding rate was 0.2% (4/2,166) in the LMWH group versus 0.1% (2/1,713) in the control group. Because of its low heterogeneity (*I*^2^ value = 0%), we used the fixed-effects model to combine the minor bleeding data. The minor bleeding rate was 6.5% (140/2,166) in the LMWH group versus 4.5% (77/1,713) in the control group. The rate of minor bleeding was significantly lower in the control group than in the LMWH group (RR: 0.64; 95% CI: 0.49–0.83; P = 0.001, Fig 8). It should be noted that in all studies, which included 3,879 patients, there were no deaths reported after knee arthroscopy.

### Sensitivity analysis

In the sensitivity analyses, the outcome of asymptomatic DVT was included because of the notable heterogeneity. To analyse the source of heterogeneity, we eliminated articles one by one. The results showed that when the articles written by Camporese et al. or Marlovits et al. were removed, the heterogeneity was significantly reduced (*I*^2^ value = 0%; RR: 0.12; 95% CI: 0.04–0.31 or *I*^2^ value = 15%; RR: 0.37; 95% CI: 0.16–0.84). Based on this result, we carefully reviewed the original research to determine the factors that caused heterogeneity in these studies. We determined that a methodological inconsistency was the cause of the heterogeneity. In the study by Camporese et al., the patients in the control group did not use LMWH to prevent thrombotic events; instead, compression stockings were used. In the study by Marlovits et al., all surgical patients during hospitalization were administered LMWH to prevent DVT, with no differentiation into groups. When patients were discharged, they were subsequently divided into LMWH and control groups. Thus, the use of LMWH before surgery or compression stockings in the control group may have had an impact on the incidence of asymptomatic DVT.

## Discussion

This meta-analysis included 7 randomized studies that assessed the efficacy and safety of LMWH in more than 3,500 patients after knee arthroscopy. In clinical research, a RCT naturally represents the gold standard. [13] A previously published study indicated that in surgical practice, a RCT could answer at least 40% of the questions surrounding clinical decisions. [14] An article published in 1995 in The Lancet showed that 53% of clinical decisions were backed by a RCT in acute internal medicine. [15] Our meta-analysis showed that the symptomatic VTE rate was 0.5% (11/2,166) in the LMWH group versus 0.6% (10/1,713) in the control group. The risk of major VTE (symptomatic VTE, symptomatic DVT and symptomatic PE) was not decreased by LMWH prophylaxis. There was no efficacy for LMWH in patients undergoing knee arthroscopy in our rearch. This finding was in accordance with the result of a recent high quality RCT[9] and meta-analysis[16].

Proximal DVT was defined when the common femoral or popliteal vein was included [6]. Therefore, the other DVT of the lower extremity was the distal DVT. To date, the clinical relevance of distal VTE is strongly disputed, and treatment is currently not recommended[17–21]. These events were not a representation of clinically relevant VTE, and distal VTE does not require treatment particularly considering the bleeding risk[22]. The NNT (number needed to treat) undergoing knee arthroscopy was 1,000 (1/(0.6–0.5%)). If the symptomatic VTE events contained distal VTE events, the NNT was 143 (1/(24/1,713-15/2,166)). We determined that the difference between these two results was substantial. Thus, the patients with distal VTE determined the outcome of the meta-analysis.As previously discussed, we excluded the distal VTE events in the symptomatic VTE events in our research.

LMWH has been extensively employed for VTE prophylaxis when undergoing moderate and high-risk surgery[23]; however, the risk of bleeding with LMWH is higher than that with aspirin. [24–25] Therefore, the complications (e.g., major bleeding and minor bleeding) after LMWH treatment cannot be ignored. The safety of LMWH in arthroscopic surgery of the knee is also concerning. In this meta-analysis, we identified a nonsignificant increase in the risk of major bleeding in the LMWH group compared with the control group, which was inconsistent with Chapelle C et al.’s[8] findings in a non-major orthopaedic setting. The study showed that the major bleeding rate was 0.2% (4/2,166) in the LMWH group versus 0.1% (2/1,713) in the control group. Moreover, for major bleeding, the NNH (number needed to harm) undergoing knee arthroscopy was 1,000. Our meta-analysis indicated significantly less minor bleeding in the control group than in the LMWH group. As previously discussed, we attributed this bleeding to the pharmacological action of LMWH. There were no deaths reported in the randomized trials included in this meta-analysis. Clinically, in general, the risk of minor bleeding did not endanger the patient’s life. Therefore, we believe that it is relatively safe to use LMWH after arthroscopic surgery of the knee.

A survey study indicated that despite the lack of a solid evidence base, most patients receive thrombosis prophylaxis when undergoing knee arthroscopy.[26] Moreover, although the substantial majority of patients received VTE prophylaxis after orthopaedic surgery, a gap between the international guideline recommendations and clinical practice was identified. [27] Our meta-analysis also showed that LMWH prophylaxis does not reduce the risk of symptomatic VTE after knee arthroscopy.

## Limitations

Several limitations cannot be neglected in this meta-analysis. First, the lack of RCTs was the most significant drawback, which increased the risk of bias in the study. Another main weakness of the lack of RCTs was the lack of correct stratification of the arthroscopic intervention. Second, heterogeneity between studies was common, such as with the asymptomatic DVT data in this meta-analysis. We performed sensitivity analyses to explain the source of heterogeneity. Third, the clinical situation of the patients and the patients’ intrinsic risk factors[28] were not included, which may have affected the studied outcomes. Fourth, the types and doses[29] of LMWH or use of different times for patients were not considered in this study. The physician experiences may have also affected the research outcomes. Therefore, more comprehensive and convincing RCTs are required to further evaluate the efficacy and safety of LMWH after knee arthroscopy.

## Conclusions

This meta-analysis showed that there was no potential efficacy of LMWH in preventing symptomatic thrombosis after knee arthroscopy compared with a control group. Studies also indicated that there was no effect on the major bleeding rate compared with the control group. The potential adverse effects of LMWH, such as minor bleeding, should be considered. Thus, LMWH prophylaxis should not be routinely considered in patients undergoing arthroscopic surgery. However, the lack of RCTs and correct stratification in knee arthroscopy in our meta-analysis may have led to selection bias. Therefore, additional multi-centred prospective RCTs are necessary.

## Supporting information

S1 Appendix PRISMA checklist. (DOC)
